# Rangatahi Youth-Led Dissemination Campaign for Cocreated Eating and Well-Being Guidelines: Process and Pilot Implementation Evaluation

**DOI:** 10.2196/71833

**Published:** 2026-04-09

**Authors:** Layla Christison, Renee Railton, Rachael Glassey, Raun Makirere-Haerewa, David Tipene-Leach, Boyd Swinburn

**Affiliations:** 1 Te Kura i Awarua Rangahau Maori Research Centre, Eastern Institute of Technology, Napier City, New Zealand; 2 School of Population Health, University of Auckland, 34 Princes Street, Auckland, 1010, New Zealand, 64 22-167-9636

**Keywords:** youth co-design, rangatahi, social media campaign, dissemination, health guidelines, youth-led campaign, health advocacy

## Abstract

**Background:**

Youth should be partners in the development and dissemination of health information created for their demographic. The Manaora Rangatahi (youth) Guidelines comprise 10 eating and 10 wellbeing messages that were cocreated with rangatahi Māori (Māori youth) in Hawke’s Bay, New Zealand, and then disseminated through a digital media campaign.

**Objective:**

This study aimed to present the process evaluation of the co-design and implementation of the pilot digital media dissemination campaign of the Manaora Rangatahi Guidelines.

**Methods:**

The 17 rangatahi who were involved in the cocreation of the guidelines codeveloped a dissemination plan, filmed video clips for each of the 20 messages, and supported the 20-week digital media campaign. The codevelopment process over 4 wānanga (workshops) is described and critiqued, and the implementation was assessed using the data analytics from Instagram and TikTok, the main social media platforms for dissemination. The rangatahi participated in a short postcampaign review survey.

**Results:**

The dissemination plan included a website for the messages, a campaign name and logo, apparel with messaging and QR codes, and the design of a digital media campaign featuring the participating rangatahi. Video clips of each of the guideline messages featuring the rangatahi were professionally developed with support from 3 Māori influencers and a video production company. The 10 phases of the campaign involved the release of 2 messages (one eating and one wellbeing) every fortnight over 20 weeks. Instagram and TikTok analytics showed that the campaign achieved >1.48 million impressions and >19,000 engagement actions (eg, likes, comments, and sharing). The mean engagement rate (Instagram 6.2%, TikTok 1.2%) was greater than or similar to the platforms’ medians across all industries (0.36% and 1.73%, respectively). Various paid promotion strategies boosted the number of impressions, and paying one of the influencers to promote the messages in phase 8 created a more than 10-fold increase in impressions on Instagram. The estimated cost of the overall campaign was NZ $125,000 (US $72,500). The majority (about 60%) of rangatahi felt the campaign was successful and engaging.

**Conclusions:**

Rangatahi have expert knowledge in how to disseminate messages to their peers. They successfully co-designed and pilot-tested the implementation of a low-cost digital media campaign using peer-to-peer messaging and videos, which achieved substantial reach. The dissemination reach was good, but was significantly influenced by paid promotions. The cost per thousand impressions was equivalent to or better than much larger government-funded health promotion social media campaigns targeting youth. The development and dissemination of eating and well-being messages aimed at youth should ideally involve partnering with the target audience to enhance the relevance and reach of the messages. A well-funded dissemination campaign with paid promotions through influencers and digital platforms could expect to achieve substantial awareness of the messages.

## Introduction

The development and dissemination of health and well-being guidelines have typically occurred through a top-down approach by government organizations with minimal input from their target audiences. This approach can be problematic, particularly if resulting materials fail to resonate with the target population and partnership approaches during guideline development and dissemination are rare [1-3].

Dissemination of health guidelines has typically been poorly funded. Websites, pamphlets, and web-based resources for professionals are a common, low-cost approach, but these are likely to have limited effectiveness in reaching young audiences. Major mass media-led health campaigns, which were well-funded, like the United Kingdom’s Change4Life [4] and Australia’s LiveLighter [5], targeting adults, can achieve widespread campaign awareness, although with small self-reported changes in behaviors [6,7]. The advent of digital media platforms has meant that dissemination campaigns using social media platforms are lower cost than paid broadcast media, can achieve wide reach, and show some promise in changing behaviors [8,9]. Youth are receptive to the use of digital health promotion [10], and campaigns targeting youth now commonly use digital platforms with promising results for increased awareness, although assessing behavior changes from such campaigns is challenging [11].

Indigenous youth face further challenges as health promotion messages and guidelines typically lack cultural relevance. Traditional knowledge and cultural connections are fundamental for the well-being of Indigenous youth [12-14], but this is not often expressed in health guidelines or dissemination pathways. In Aotearoa New Zealand, there are consistent disparities in health outcomes between Pākehā (NZ European) and Māori populations [15]. Eurocentric, biomedical approaches to health communication are doing little to reduce these disparities. Attempts to design health interventions for Māori rarely extend to partnership, let alone full design by Māori. Alternative, particularly Indigenous, interpretations of health and wellbeing are uncommon [16].

The co-design of health communications to inform healthy behaviors has led to an increased relevance and uptake by the intended end users [17]. Further, health communication campaigns are more successful if they include the cultural characteristics, belief systems, life experiences, and values of the target audience [18,19]. This approach enhances audience receptivity, message acceptance, and salience of information, leading to increased audience buy-in [20,21]. Commentators note that the active involvement of target audiences in the planning and creation of health messaging increases the inclusion of cultural characteristics and may also allow capitalization of pre-existing social networks [20,22].

In NZ, healthy eating guidelines for youth were initially developed by the NZ government in 2012 and updated in October 2021 [23]. Although web-based resources and pamphlets relating to these exist, they are not actively disseminated. Similarly, wellbeing guidelines for youth covering areas such as physical activity and sleep have been developed but are not actively disseminated [24]. Such passive approaches to dissemination mean that the impacts of these resources on the health and well-being of NZ youth are unlikely to be of any significance.

Social media platforms, such as TikTok and Instagram, have become integral to youth communication and information-seeking, with 87% of teens using these platforms to seek health information [25] and 37.8% of teens seeking information on fitness and nutrition [26]. These platforms offer unique advantages for health communication, including peer-to-peer interactions that enable knowledge exchange within established social circles. This positions the audience as active participants in the dissemination process, rather than passive recipients [20,22,27,28].

Studies are needed to address these shortcomings by working with youth to co-design messages with cultural relevance and meaning and disseminate them on the social media platforms youth already engage with and using peer-to-peer networks.

Acknowledging the need for culturally relevant and youth-created health guidelines, the Manaora Rangatahi Eating and Wellbeing Guidelines (Manaora Guidelines) project was developed with the aim of improving upon the current NZ Ministry of Health nutrition [23] and physical activity [24] guidelines for youth, especially rangatahi Māori (Māori youth). The project was in 2 parts: the cocreation of a set of eating and wellbeing guidelines, and the cocreation and implementation of a dissemination plan for these guidelines targeting youth.

**Figure 1.**
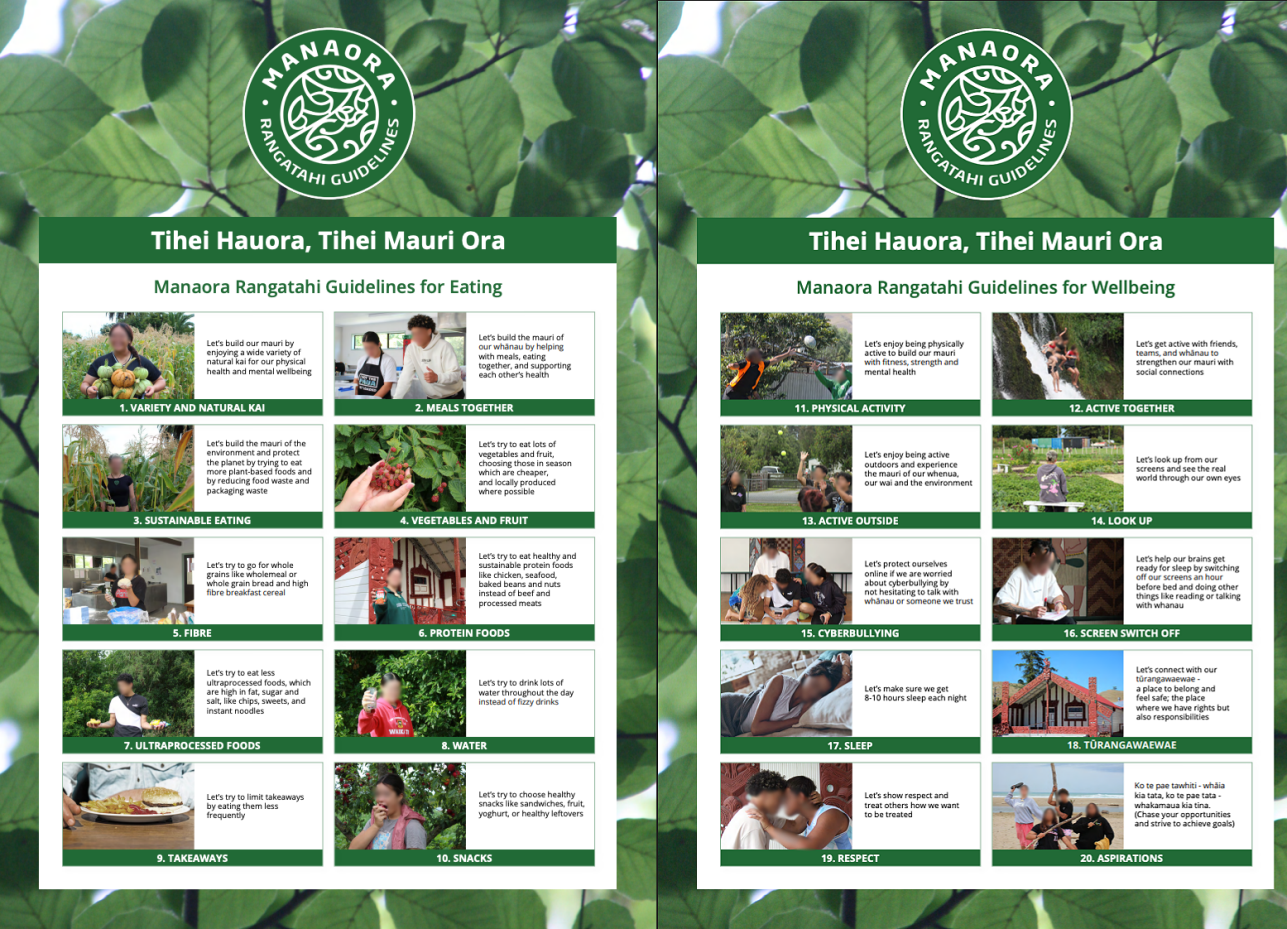
The Manaora Rangatahi Guidelines for eating and well-being. Mauri is life force, whānau is family, tūrangawaewae is a place to stand.

The Manaora Guidelines (Figure 1) were cocreated with 17 rangatahi Māori from 4 schools in Hawke’s Bay, NZ, over several wānanga (workshops) held at a marae (traditional Māori meeting place). The approach used ensured that rangatahi were positioned as active creators rather than passive recipients of the health messages, using both scientific evidence and mātauranga Māori (traditional Māori knowledge) in the guideline creation. The resulting rangatahi-created guidelines consisted of 20 messages (10 eating and 10 wellbeing) that use invitational rather than prescriptive language (eg, “Let’s try to…) and acknowledge the journey that young people may face in adopting the guideline messages. A distinguishing feature of these guidelines is their integration of Te Ao Māori (Māori worldview) concepts, as shown by the incorporation of terms like mauri (life force), whānau (family), and tūrangawaewae (a place to stand) into several messages and the use of te reo Māori (Māori language) throughout. These guidelines further extend traditional health recommendations by addressing contemporary concerns such as sustainability, digital well-being, and social connections and end with an inspirational whakataukī (proverb). The guidelines were peer-reviewed by 94 rangatahi in structured feedback sessions, and the full process of the creation of the guidelines is described elsewhere [29].

The aims of this paper are to present the process evaluation of the codevelopment and implementation of a pilot digital media campaign developed to disseminate the Manaora Guidelines. In other words, how well did the partnership approach with youth work, and what level of engagement through Instagram and TikTok could the campaign achieve?

## Methods

### Overview

The 17 rangatahi who cocreated the Manaora Guidelines (Figure 1) participated in this dissemination component. Principals from Hawke’s Bay schools with high Māori student rolls (n=15) were invited to offer Year 12 students (16 to 17 year olds) the opportunity to participate in this rangatahi guidelines project. A total of 17 students (6 males, 11 females, 16 Māori, 1 non-Māori) from 4 schools agreed to participate in the cocreation of the guidelines [29].

This paper presents the dissemination plan and digital media campaign, which were developed in stages over 4 wānanga with the same 17 rangatahi who cocreated the guidelines. The first stage involved the co-development of the dissemination plan. Stage 2 consisted of the creation of materials, including the shooting of the videos for each message. Stage 3 involved the implementation of the digital media campaign, which was launched in March 2024 with a 2-week run-in period and 20 weeks of the campaign. Stage 4 involved a postcampaign review. All wānanga were held at the Houngarea marae in Hawke’s Bay.

### Planning

The first stage was a wānanga held over 3 days in November 2023. The dissemination planning process consisted of a series of presentations by senior researchers on the basic principles of social marketing and health and well-being message dissemination for personal and community benefits. Exemplars of various campaigns were presented, and aspects of each campaign that generated audience engagement and influenced consumer perception of a product or behavior were discussed. The planned nature of a communications campaign was also discussed, including the need to be very clear about the objective of the campaign, the strategies used, the style and “feel” of the delivery, the nature of the messages, and the measures of success.

While social marketing is underpinned by many theoretical models, the project colead (BS, public health physician) focused primarily on the exchange theory [30] and the stages of change transtheoretical model [31]. Commercial marketing, with which participants were very familiar, was explained in terms of its basic 4P model (product, price, placement, and promotion), and then, using existing campaigns as a guide, the parallels with social marketing were made. Exercises identifying the “exchange value” in the “products” of both commercial and social marketing helped to show how these exchanges needed to be explicit. Indeed, the structure of the wording in the guidelines made these exchanges explicit. For example, “Let’s build the mauri (life force) or our whānau (family) by helping with meals, eating together, and supporting each other’s health” encompasses both the costs (helping out) and the social and health benefits. An exercise reflecting on their own willingness to enact some of the guidelines showed that many rangatahi were in the early stages of change.

The participants had considerable lived experience of social media platforms and understood the potential for viral peer-to-peer communication and influencer boosting to disseminate messages. The concepts and theories of social networking as they apply to marketing are well articulated, although the characteristics of “virality” are still being uncovered [32].

The process used allowed the rangatahi to incorporate mātauranga Māori (traditional Māori knowledge) into the guidelines and the dissemination and implementation plan. Project colead (DTL) has extensive knowledge of mātauranga Māori, and he guided the participants on these issues throughout the original development of the guidelines and this process of dissemination planning and implementation. The rangatahi were all familiar with many aspects of tikanga Māori (Māori processes), and they led many of the aspects of the work. For example, they created the name of the guidelines, conceptualized the Māori images for the logo and branding of materials, came up with many of the ideas for the videos, and starred in all the video clips.

Small group discussions were used to brainstorm potential dissemination methods from the rangatahi. A follow-on discussion covered the campaign branding and content style of a digital media campaign. By the end of this wānanga, a draft dissemination plan had been developed, including an outline of a digital media campaign, including video outlines.

### Development of Materials

The draft outlines for each video developed at the wānanga were converted into scripts through discussions between the research team and the local Māori production company used to produce the videos. The scripts were also circulated to the rangatahi group for their feedback. A further 3-day wānanga was held in January 2024, focusing primarily on the creation of video content for the social media campaign. Three Māori social media influencers suggested by the rangatahi group, and popular with youth in NZ, were contracted to attend the wānanga and participate in the filming. These influencers had a combined reach of more than 385,000 accounts across TikTok and Instagram, with professional backgrounds ranging from full-time content creation on social media platforms to mainstream television hosting of Māori-centered programs.

Over the 3 days, the rangatahi, influencers, production team, and research team reviewed and revised the scripts and altered filming plans to fit the visions of the rangatahi. Collaboration between researchers, rangatahi, influencers, and filmmakers during this process ensured meaningful and relevant video materials for the campaign. The filming of the 20 messages from the Manaora Guidelines occurred at the marae and at various well-known Hawke’s Bay locations. The Māori production company did the filming and postproduction of the videos for the campaign.

### Implementation and Dissemination

The third stage consisted of the digital media campaign as devised by the rangatahi. This was implemented over a 22-week period running from February to July 2024. Further details on the campaign can be found below. The degree of dissemination was assessed using the data analytics provided by Instagram and TikTok. In particular, the main indicators were “impressions,” which is how many times the video was viewed or partly viewed, and “engagement rate,” which is the number of engagements divided by the number of impressions, expressed as a percentage. Instagram uses the term “interactions” which is akin to TikTok’s “engagement,” both meaning actions such as liking, commenting, sharing, downloading, and clicking on links.

### Postdissemination Review

The fourth stage was held on one day in July 2024 and consisted of a review of the digital media campaign after its completion and allowed the rangatahi to reflect on their experience being part of this project. A questionnaire was developed (Multimedia Appendix 1) to allow the rangatahi to rate their engagement with the campaign and the campaign messages, as well as their peers’ engagement with the campaign. Two open-ended questions were included to seek feedback on improving the reach and overall campaign. The survey was delivered via electronic tablets using Alchemer software (Alchemer LLC).

### Ethical Considerations

Ethics approval was received from the University of Auckland’s Auckland Health Research Ethics Committee (AH26095) and the EIT Ethics Committee Ethics Committee (EA04270123). Informed consent was obtained from each participant and their parent/caregiver before attending the wānanga. This consent included permission to use participant images and video footage as part of the campaign. The rangatahi were given a small koha (gift) at the end of the campaign as a token of their many days of contribution to the project.

## Results

### Planning

The dissemination plan suggested by the rangatahi centered on the use of social media to reach their target audience, specifically choosing Instagram, TikTok, and YouTube as primary channels. This was based on their understanding of peer communication and identifying their own ability to capitalize on pre-existing social circles on these platforms. The campaign strategy prioritized youth voice and experience and extended the reach by using established Māori social media influencers. All decisions were made collaboratively, with all participants having a say. There were no major points of contention and substantial issues, such as the naming of the guidelines, were decided by consensus, as is the custom on the marae.

### Campaign Materials

The Manaora branding and logo evolved through collaborative discussion, with the rangatahi initially opting for “Tihei Hauora” (be alert to your health) as the campaign name. This was later replaced by a different name that the group felt better encompassed their kaupapa (mission). Thus, the group ultimately landed on the invented name “Manaora”—a blend of the words “mana” (prestige) and “ora” (health). The campaign’s visual logo was inspired by their environment, featuring the kōwhaiwhai (Māori decorative design) planting pattern of a local community garden visited by rangatahi during the wānanga, and was one of the locations featured in the filming of campaign content.

The campaign strategy focused on creating 20 short videos explaining each of the 20 guideline messages and a 90-second trailer. The rangatahi envisioned an interactive and conversational style to the message videos, relying heavily on humor to capture and maintain audience attention. The rangatahi decided that, alongside the influencers, they should also appear in the videos due to their established knowledge on the guidelines and recognition of the importance of peer representation. These videos can be found on YouTube [33]. Supporting materials included branded merchandise (hoodies featuring QR codes linking to the Manaora webpage), comprehensive wellness summaries, and detailed guideline explanations. Table 1 displays the details of the campaign dissemination plan.

**Table 1.** Campaign dissemination plan.

Campaign components	Key details
Objective	To disseminate the Manaora Rangatahi Guidelines messages to as large a peer audience as possible
Media	Social media platforms: TikTok [34], Instagram [35], YouTube [33] Manaora Rangatahi Guidelines webpage [36]
Target audience	All New Zealand youth aged 12 to 24 years, especially Māori youth.
Content	Social media video clips 20 videos (50‐70 s focusing on each of the 20 Guideline messages) 90 s trailer video Featuring NZ Māori influencers
Strategies	Launch event Release 2 guideline messages and their accompanying videos per fortnight over a 20-week period (ie, 10 campaign phases) Branded merchandise for use as incentive to target audience for engagement with the social media campaign
Branding	Name and logo development Hoodies (featuring logo, tukutuku [ornamental latticework] panel design from the marae that hosted the wānanga series, and QR code linking to the Manaora Instagram account)
Materials	Eating and Wellbeing Interactive Guidelines (comprehensive overview of eating and wellbeing messages including links and QR codes to additional resources and social media pages) [37] Comprehensive Wellness Summary (summary of the 20 messages, for quick reference or large display poster) [38] Eating and Wellbeing Explained (detailed explanations and references of each of the 20 messages) [39] Posters, each spotlighting one of the 20 key messages [40] The Roots of Our Logo (the inspiration and story behind our logo) [41]
Measures of reach	Social media analytics (Instagram and TikTok)—Impressions and Engagement rate

### Implementation

The Manaora Rangatahi Guidelines social media campaign was launched at another local marae, Waipatu, and attended by school representatives, health and community stakeholders, local Iwi (tribe) representatives, and local and national media. The rangatahi spoke about the importance of the project, highlighting their increased connection with Māoritanga (Māori cultural practices and beliefs) through the project. During the launch event, the campaign trailer and the first 2 of the 20 guideline message videos were screened to the audience.

The campaign (referred to as the Manaora campaign) ran over 22 weeks—a 2-week lead-in and 10 fortnightly phases where 2 campaign messages (one eating and one wellbeing) were released in each phase across the selected social media platforms. An additional 32 infographic resources providing background information on the guideline messages and a series of “behind the scenes” posts that showcased the filming process were also posted to Instagram. During the campaign, 4 rangatahi who had expressed interest were granted access to the social media accounts to create additional content to increase engagement.

Various promotion strategies were tried throughout the campaign, with the small promotion funding available. During the 2-week precampaign period and the first 3 phases (ie, 6 weeks) of the Manaora campaign, the paid promotion on TikTok was directed at increasing campaign awareness. This means that the platform’s algorithm targeted user views of the video content, aiming to amplify the number of individuals who saw the content. However, the engagement of video content was low during this period—the audience was seeing the messages but not necessarily watching the whole video or engaging with the content through likes, comments, saves, or shares.

Therefore, the objective of the paid promotion for TikTok was changed to target engagement for phases 4‐8. This resulted in fewer overall views per video, but increased content interaction and user engagement. Paying for promotions to enhance either impressions or engagement clearly works. Although the overall awareness was impacted due to the change in advertising objective, meaningful interaction increased, which means that individual users spent more time watching the messages and were more likely to follow the Manaora account and engage with content throughout the campaign’s entirety.

Paid promotion was used for Instagram to “boost impressions” but only for the run-in fortnight and the first fortnight (phase 1) of the campaign. At phase 8, we contracted one of the Māori influencers who was involved in the filming of the videos to make her own video clip of the campaign and her involvement with it, and to post it on her Instagram and TikTok accounts in partnership with the Manaora campaign.

**Figure 2.**
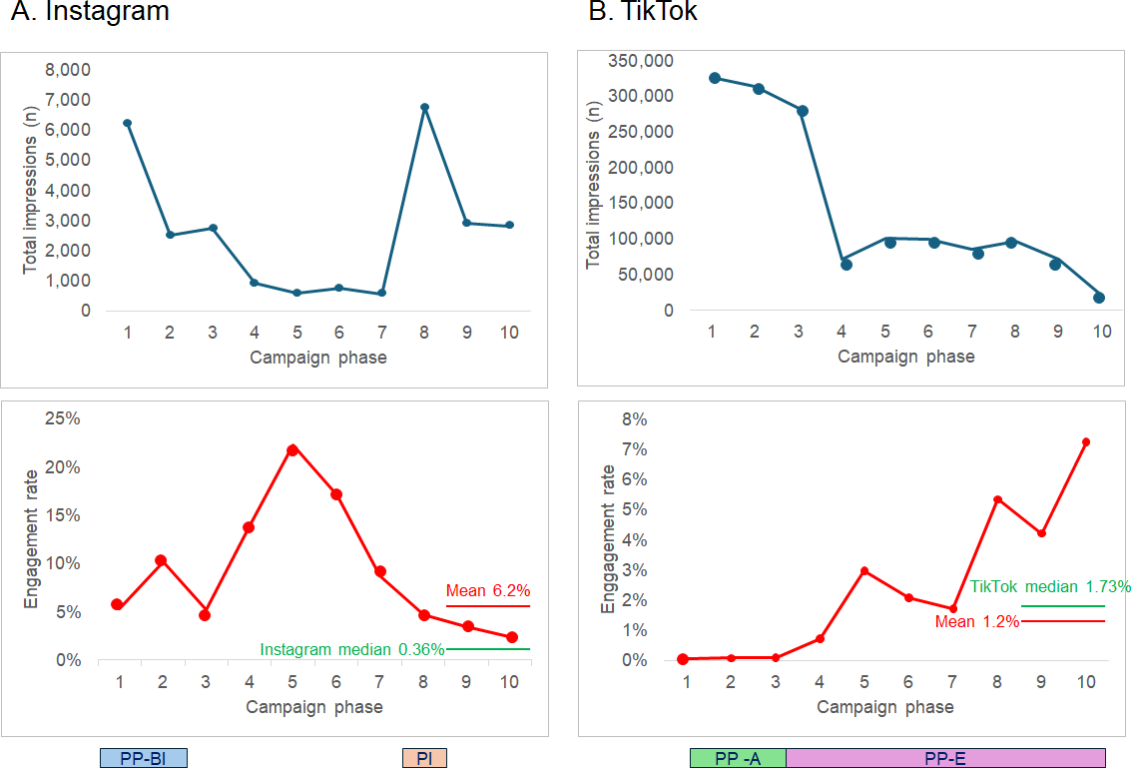
Instagram and TikTok analytics by the 10 fortnightly campaign phases. Total impressions (number of views) in blue and engagement rate (engagements/impressions) in red. Mean engagement rates for the campaign are in red and median rates for Instagram and TikTok across all industries are in green. The different promotion activities tried during the campaign are shown for both platforms. PP: paid promotion; PI: paid influencer; PP-A: paid promotion-awareness; PP-E: paid promotion-engagement.

Social media analytics on the impressions and engagement of the campaign content were downloaded weekly across the social media platforms (Table 2 and Figure 2). TikTok’s algorithm typically generates higher impression counts due to its “For You Page.”

**Table 2.** Summary of user interaction analytics for the social media platforms across the 10 phases of the campaign. Note that Instagram uses the term “interactions” for engagement. Medians for the platforms are across their 14 industry categories [42].

Platform and metric	Values
Instagram	
Impressions, n	26,916
Engagement, n	1661
Engagement rate (%)	6.2
Median platform engagement rate (%)	0.36
TikTok	
Impressions, n	1,455,643
Engagement, n	17,479
Engagement rate (%)	1.2
Median platform engagement rate (%)	1.73

A breakdown of the audience age group was available from the TikTok analytics, and it showed that 20% of the target audience was aged 13-17 years, 50% of the target audience was aged 18-24 years, and 30% of the target audience was aged 25-34 years. The campaign achieved substantial, but highly variable, reach across the platforms over the dissemination duration (Figure 2). Since impressions are the denominator for the engagement rate, there is a clear reciprocal pattern between the 2 with higher engagement rates seen during low impressions periods. Instagram impressions started at around 6000, decreasing to under 1000 per fortnight over phases 2‐7 when there were no paid promotions. The paid influencer promotion in phase 8 resulted in a 10-fold boost in impressions on Instagram, with some hangover effects into the last 2 phases of the campaign. On TikTok, the added impressions from the influencer promotion made up for the loss of impressions through the ending of the paid promotions through the platform, but these rapidly tailed off in the last 2 phases where there was no paid promotion.

TikTok had over 50 times the number of impressions compared with Instagram, and these were especially high (about 300,000 impressions per fortnightly phase) over the first 3 phases when there was paid promotion for achieving increased awareness. With the switch to paid promotion for engagement, this indicator increased over the rest of the campaign. While the mean engagement rate for the campaign fell below the TikTok median rate, it is clear from Figure 2 that, as one marker of the success of a campaign, it is highly influenced by the level of paid promotions for engagement and the reciprocal relationship with the number of impressions.

The total costs for the digital media campaign came to around NZ$ 125,000 (US $72,500). The largest cost, accounting for 32% of the cost, was personnel costs for a research assistant who managed the implementation of the digital media campaign. The creation of the videos, which included on-site professional filming over 3 days and postproduction editing, accounted for 22% of the cost. Social media influencer engagement accounted for 15% of these costs, covering their participation in the wānanga, involvement in the filming, and subsequent content creation by one of the influencers. TikTok advertising costs and the branded apparel accounted for 4% and 3% of these costs, respectively. The remaining costs included venue hire, catering, koha (donations), and travel expenses for the wānanga series.

### Postdissemination Review

The postcampaign evaluation showed generally positive perceptions from the rangatahi on how the campaign had performed. Of the 17 rangatahi, 10 rated the campaign as successful or very successful, while 7 were neutral, and one viewed it as very unsuccessful. Engagement with the campaign was also mostly favorable, with 11 rangatahi reporting they were quite or very engaged with the campaign, while 6 felt somewhat engaged, and one was not engaged at all. Engagement of peers with the campaign was rated as successful or very successful by 10 of the rangatahi, while 7 rated it as neutral and one as very unsuccessful. The 2 open-ended questions provided a variety of qualitative feedback, but on balance, this was not considered rich enough to warrant a formal analysis.

## Discussion

### Principal Findings

This study evaluated the process of developing and implementing a dissemination strategy for the Manaora Guidelines with the rangatahi who cocreated the original guidelines. With guidance and professional input, the rangatahi developed an innovative digital media campaign, which included the creation of a substantial amount of campaign material. The campaign of 10 phases over 20 weeks achieved engagement rates that were much higher than industry standards for Instagram and about the industry standard for TikTok.

Achieving nearly 1.5 million impressions and more than 19,000 engagements was very positive for a low-cost campaign. The added impacts of paid promotion through the platforms and the influencer were clear in the data analytics. With a greater investment in promotions, the reach and engagements could have been considerably greater; however, as with all digital media campaigns, assessing the conversion of awareness of the messages to attitudinal and behavioral changes is a big challenge [11] requiring a more comprehensive evaluation than just the platform analytics.

While TikTok showed a much higher reach, Instagram had a 5 times higher engagement rate, and this may in part be due to the additional static content we posted, such as “behind the scenes” images from the filming process, simplified infographics, and “top tips” video content focusing on different ways to enact the guidelines. These were posted only on Instagram because these formats are not suited to the TikTok platform.

### Partnering With Youth

The codevelopment process worked very well, and the rangatahi worked hard over several days and enjoyed the creative processes. However, interestingly, they did not universally rate the campaign as a success and gave informal feedback that they felt they could have worked harder on the dissemination campaign by being more actively engaged online.

By partnering with youth, in this case Indigenous youth, in the creation and sharing of health information that was relevant and culturally appropriate, the campaign was more likely to reflect the belief systems, life experiences, and cultural values that would be relevant to that particular target audience [43] and arguably, to the wider youth audience as well, although we have no direct evidence of this. This process acknowledged that rangatahi are experts in the types of messages and delivery that are most likely to engage them and their peers [44]. Our findings extend on this by showing that, with appropriate guidance, rangatahi are well able to be full partners in all steps of health communication, from message development through to implementation and dissemination strategies. As such, they should be involved in guideline development and dissemination as genuine partners, and not just as participants; however, more formal evaluations of impact would be needed to demonstrate these assumptions. While other studies have collaborated with youth to co-design social media campaigns [45], few have involved youth in the implementation of the dissemination.

Ensuring all aspects of this project used a Te Ao Māori lens was vital throughout this project. This allowed the rangatahi to connect with the project and incorporate mātauranga Māori into the guidelines to make them relevant for themselves and their wider peer group. This was evident at all stages of the project, from the use of Māori concepts, such as mauri (life force), tūrangawaewae (a place to stand), whakatauki (proverbs), and te reo Māori (the Māori language), to the involvement of Māori influencers and locations of significance for filming. This allowed the development of a dissemination strategy that was innovative and based on cultural identity.

The co-creation of a dissemination plan was enriching on multiple levels beyond that of just the campaign outcomes. For the rangatahi, it affirmed their ideas and knowledge and positioned them as experts of their own world. The process allowed for the creative generation of innovative ideas and enhanced collaboration as they learned to work in teams with each other and alongside research and communications experts. This process not only improved the relevance and method of health message dissemination for youth in general, but also had positive effects on the partnered youth themselves, as can be seen in their reflections of their own experiences (Voices of Manaora: Unveiling our messages [46], and Manaora Reflections [47]) and what they gained from their involvement, potentially improving their health-related attitudes and beliefs.

### Campaign Lessons

Twenty messages are a large number to include in any public messaging campaign. It was decided to include all the messages because they had all been co-created by the rangatahi. National dietary guidelines, including the ones used as exemplars for the cocreation of the rangatahi guidelines (Brazil, Chile, Mexico, Norway, US, NZ), typically have about 10 messages [48]. Added to that, the rangatahi included well-being guidelines for physical activity, sleep, bullying, screen time, respect, and psychological well-being. Fewer messages could potentially be used with higher impact in other circumstances, such as in nutrition curricula.

There are several lessons learned from this study. First, with guidance from public health professionals and influencers, a group of rangatahi was able to cocreate innovative and engaging material for a dissemination campaign. Both the academic concepts of social marketing and the lived experience of the rangatahi were influential in developing the plan. However, in the implementation phase, the participants themselves could have been more actively engaged online in commenting or sharing their thoughts and experiences on the messages as they were posted every fortnight. Second, it was difficult to judge the success of such a campaign just on the platform analytics. Understanding how well the messages were received and whether they resulted in a shift in the stage of behavior change would require a much more extensive evaluation. However, it would only be plausible to postulate changes in behavior if the campaign were more extensive, ongoing, and linked to other strategies, such as inclusion of messages in the curriculum and dissemination through the education and health systems. Third, the development and dissemination of the Manaora Guidelines was part of a research project, rather than a government-led exercise. This meant that there was no ongoing implementation pathway for the guidelines—a disappointing end to such an innovative project.

### Implications

The implementation of the Manaora Rangatahi Guidelines dissemination plan was low-cost and did achieve meaningful engagement of almost 1.5 m impressions. While other youth-directed digital media campaigns relied on paid advertising and professional expertise, as we did, few outlined their direct campaign costs. Kearney et al [49] achieved over 3.1 million impressions using social media experts and paid advertising to combat the racial marketing of unhealthy foods, but the cost of implementing their campaign was not provided. Weir et al [50] did a cost analysis of a campaign (FinishIt) aiming to prevent youth smoking in the United States over a 3-year period. They concluded that the program was cost-effective, but the total cost of that campaign was very high at US $162 million. Similarly, Sim and Wong [51] evaluated youth engagement with a mental health campaign promoted on social media (CHAT) and found increased audience engagement with increased social media advertising, but they did not provide campaign costs. Thus, future research should include campaign costs as well as engagement rates to allow for the cost-effectiveness of health campaigns to be established. Further research is also required to explore how best to balance the organic promotion of campaign content with strategic paid influencer and platform promotions.

The role of digital media in disseminating health information is substantial. Hawke et al [28] showed that social media with infographics and videos was important in the dissemination of COVID-19 health information to youth, and Kostygina et al [52] showed that social media influencers and memes can engage youth populations by making health messages more appealing and accessible. Digital engagement also allows campaigns to target audiences, which is crucial for addressing health disparities. Our study reinforces all these findings.

While the Manaora campaign achieved substantial reach through organic youth-led promotion and minimal advertising, our use of established influencers with pre-existing follower bases increased this reach further. Influencer costs vary based on their following size, engagement rates, and campaign requirements and need to be considered within the campaign budget. Thus, policymakers and health promotion organizations need to consider funding for digital media campaigns that incorporate both youth partnerships and strategic influencer and platform promotions.

Finding similar low-budget social media campaigns in New Zealand to compare analytics is difficult. Most health promotion campaigns that are run through social media and which have published analytics are government-funded. Their budgets, reach, and impact are all much greater than the present campaign. For example, the campaign “Love better” addressing domestic violence and targeting youth cost over NZ $10 million (US $5.8 million), ran over 3 years, achieved 4.25 million and 1.6 million “engagements” (views, reactions, comments, shares, saves) for TikTok and Meta (Instagram and Facebook) respectively. This gave engagement rates of 22% and 8% and cost per thousand views of NZ $2.75 and $3.97 (US $1.597 and 2.30) respectively [53]. The costs per thousand views in this study were NZ $0.09 (US $0.0522) for TikTok and $4.64 (US $2.69) for Instagram, showing equivalent or better cost-effectiveness.

The dissemination of the Manaora Rangatahi Guidelines through social media platforms has significant potential for wider reach and transferability. While message dissemination was successful through digital media, the model developed here has broader implementation opportunities, particularly through schools, community organizations, and health providers. Furthermore, the transferability extends beyond just that of the guidelines themselves to the youth partnership methodology that was used here, which could be scaled from local to national levels. Involving the secondary audiences, such as educators and community health workers, in the creation of the dissemination plan, alongside the rangatahi, would ensure the resources created were also fit for purpose for next-users to include in their own programs across different contexts. The findings of this study strongly suggest that governments, health, and community organizations should partner with their target audiences, particularly youth, in the development and dissemination of future guidelines regarding health and well-being messaging.

### Strengths and Limitations

The strengths of this study include the high level of engagement, ownership, and innovation shown by the rangatahi. The rangatahi involved in this project remained engaged throughout the year-long process of collaboration in the development, implementation, and postevent evaluation. This highlights the strength of this method for participation retention as well as health communication messaging and dissemination. In addition, this process highlights a potentially low-cost but effective means of dissemination in a target audience that can be notoriously difficult to reach by conventional communication channels.

Limitations include the small number of rangatahi involved in the process, which may limit the generalizability of the findings or the reach of the dissemination campaign. Also, as noted, the participants could have been more actively engaged in amplifying the messages as they were released fortnightly. While the cocreation approach worked well, the pathways to scalability, sustainability, and enhancement of dissemination by participants were not evident. These are important questions for future research. While the use of influencers as message amplifiers was used in the development of materials and bolstering reach at the end of the campaign, their involvement did come with added costs. The cost-benefit of using influencers for social marketing of healthy eating and well-being messages remains an open question. We do not know whether the exposure to these youth-developed messages through the campaign resulted in behavioral or attitudinal changes in the target population, making the behavioral effectiveness of the campaign unclear. The lack of more appropriate platform benchmarks than the “all industries” medians limits the comparability of this study to other youth-focused campaigns. In addition, the sustainability of campaigns needs to be further explored to maintain campaign relevance. Rangatahi were not fulsome in their qualitative feedback on the campaign, thus limiting the richness of this aspect of the evaluation. Finally, the rangatahi were not involved in the administrative aspects of the campaign management or promotion of content, both of which are important components of any social media campaign.

Involving next-users in the dissemination would also allow for more independent feedback, assessment of structural impacts (eg, incorporation into curricula), and perhaps behavioral changes. The messages were not independently pretested before dissemination, which was a limitation, and if the guidelines were to be formally included in school curricula, they would need further evaluation for suitability for that purpose.

### Conclusions

This study highlights the value and importance of youth partnership in the cocreation and subsequent dissemination of health guidelines. The success of the Manaora Rangatahi Guidelines campaign offers a model for future youth-centered health initiatives, emphasizing the importance of genuine partnership with target audiences to develop dissemination strategies that are relevant, engaging, and culturally appropriate in order to achieve greater uptake.

## Supplementary material

10.2196/71833 Multimedia Appendix 1 Glossary of the Māori terms and the survey questions used.
