# Supplementary material for: Rangatahi Youth-Led Dissemination Campaign for Cocreated Eating and Well-Being Guidelines: Process and Pilot Implementation Evaluation
Source: JMIR Form Res. 2026 Apr 9;10:e71833. doi: 10.2196/71833 (PMC13064959; doi:10.2196/71833)
Supplement: Multimedia Appendix 1 [file formative-v10-e71833-s001.docx]

## Glossary

Glossary: Te Reo Māori terms in the manuscript. Definitions adapted from Te Aka Online Māori Dictionary

| **Māori Term** | **Explanation** |
| --- | --- |
| kaupapa | mission |
| koha | donation |
| kōwhaiwhai | Māori decorative design |
| mana | prestige |
| Māoritanga | Māori cultural practices and beliefs |
| marae | meeting house |
| mātauranga Māori | traditional Māori knowledge |
| mauri | life force |
| noho | overnight stays |
| ora | health |
| Pākeha | New Zealand European |
| rangatahi | youth |
| te ao Māori | Māori worldview |
| te reo Māori | Māori language |
| tukutuku | ornamental latticework |
| tūrangawaewae | a place to stand |
| wānanga | workshop |
| whakawhanaungatanga | the making of relationships |
| whakatauki | proverb |

## Appendix A: Post campaign questionnaire

#### How would you rate your engagement with the social media campaign online?

( ) Not engaged at all ( ) Somewhat engaged ( ) Quite engaged ( ) Engaged ( ) Highly engaged

#### How would you rate your engagement with the messages of the campaign?

( ) Not engaged at all ( ) Somewhat engaged ( ) Quite engaged ( ) Engaged ( ) Highly engaged

#### How would you rate the success of the social media campaign engaging with your friends or peers at school (for example, people interacting with posts or talking about the messages)?

( ) Very unsuccessful ( ) Unsuccessful ( ) Neither successful nor unsuccessful ( ) Successful ( ) Very successful

### What ideas do you have for ways to get the messages out more widely to other rangatahi?

### What could have been improved with the campaign and/or what parts did you really like?
